# The first complete mitochondrial genome of genus *Phytomia* (Diptera: Syrphidae)

**DOI:** 10.1080/23802359.2020.1780976

**Published:** 2020-06-17

**Authors:** Juan Li, Hu Li

**Affiliations:** Shaanxi Key Laboratory of Bio-resources, School of Biological Science and Engineering, Shaanxi University of Technology, Hanzhong, China

**Keywords:** Hoverfly, mitogenome, phylogeny

## Abstract

The complete mitochondrial genome (mitogenome) of *Phytomia zonata* was sequenced in this study, it is the first complete mitogenome in the genus *Phytomia*. The wholly sequence is a circular DNA, and with 15,716 bp in length including 37 typical genes. No gene arrangement was observed in this sequence. The common start and stop codons were ATN and TAA, respectively. A phylogenetic tree was generated based on dataset of 19 complete mitogenomes from hoverflies and two Lauxaniidae species which as outgoup, and shows *Phytomia* is closer to *Eristalis*, supports the monophyly of Syrphinae.

The hoverfly species, *Phytomia zonata* (Fabricius, 1787) (Syrphidae: Eristalinae), is widely distributed around the world. It is a bumblebee mimic possessing clear black-yellow color pattern, and can be easily recognized by the sturdy body with thick hairs, vein r_4 + 5_ with a loop stretched into the closed cell R_1_, the plumose aristae at basal half, and the abdomen segment 3 and 4 with a pair of yellowish-brown narrow fasciae on their anterior margins (Huo et al. 2007; Huang and Cheng 2012). The adults of *Phytomia zonata* can be found flying around the flowers of Theaceae and Ericaceae, such as *Camellia oleifera* L. and played an important role of insect pollinator like other hoverflies (Yokogawa and Hotta 1995; He et al. 2010).

Up to now, only 14 complete mitochondrial genomes (mitogenomes) have been reported against comparing with the large number of Syrphidae (Li et al. 2017; Pu et al. 2017; Li 2019; Sonet et al. 2019; Li and Li 2019; Chen et al. 2020; Yan et al. 2020). In this study, we sequenced the complete mitochondrial genome of *Phytomia zonata* (Fabricius, 1787) (GenBank accession number: MT478107).

Specimens of *Phytomia zonata* were collected from Huayang National Nature Reserve, Shaanxi Province, China (107°58′23″E, 33°35′ 48″N) in July 2018. Specimens were immersed in absolute ethanol and stored at −20 °C. Genomic DNA extraction was using a TIANamp Genomic DNA Kit (Tiangen, Beijing, China) and PCR amplification using a Taq PCR Master Mix (2×, with Blue Dye) (BBI Life Sciences), experimental operations were strictly carried out according to the manufacturer’s introduction. Voucher specimens were deposited in the Museum of Zoology and Botany, Shaanxi University of Technology, Hanzhong, China (SUHC) (accession number of the specimen for sequencing in this study is 201902-1).

The complete mitochondrial genome of *Phytomia zonata* was sequenced under Illumina NovaSeq6000 platform and assembled with Geneious Prime (Kearse et al. 2012). Protein-coding genes (PCGs) were identified by blasting with those of similar species in NCBI, transfer RNA genes (tRNA) were identified with ARWEN v1.2 (Laslett and Canback 2008), ribosomal genes (rRNA) were determined by the boundary of tRNAs and aligned with referred species. Control region was confirmed by the boundary of tRNAs.

The complete mitogenome of *Phytomia zonata* (Fabricius, 1787) was 15,716 bp in length with highly AT biased of nucleotide composition (40.9% of A, 38.5% of T, 8.5% of G, 12.2% of C). All 37 typical mitochondrial genes were present in this sequenced species including 13 PCGs, 22 tRNAs, 2 rRNAs and control region. No gene arrangement was found in this sequence, the characteristic of *Phytomia zonata* mitogenome was similar with *Drosophila yakuba* (Clary and Wolstenholme 1985). Except *ND1* started with TTG, remaining 12 PCGs were using ATN as the start codon (*ND2*, *COX1*, *ND3*, *ND5* and *ND6* used ATT, *COX2*, *ATP6*, *COX3*, *ND4*, *ND4L* and *Cytb* used ATG, *ATP8* used ATC). As for stop codons, *ATP8* and *ND3* were ended with an incomplete T and TAG respectively, others were used TAA as the stop codon. The 16 intergenic spacers were ranged from 1 to 34 bp, the 11 gene overlaps were 1 to 16 bp.

Phylogenetic tree was constructed based on the whole mitogenome sequence of 19 Syrphidae species and 2 Lauxaniidae species as outgroup (https://www.ncbi.nlm.nih.gov/) using the Maximum Likelihood (ML) method with 1000 bootstrap replicates by MEGA 6.06 (Tamura et al. 2013) (Figure 1). The result supports Syrphinae is a monophyletic group, it agrees with previous studies (Li 2019; Li and Li 2019; Sonet et al. 2019; Yan et al. 2020). Eristalinae monophyly is unclear because species were not clustered together. The *Phytomia*, which blongs tribe Eristalini, is closer to *Eristalis*. The monophyly of Milesiini was not supported. The phylogenetic relationship among Syrphidae was (((((*Eristalinus* + *Eristalis*) + *Phytomia*) + *Helophilus*) + *Syritta*) + * Volucella*) + (*Korinchia* + (*Melanostoma* + (*Ocyptamus* + (*Eupeodes* + (*Simosyrphus* + *Episyrphus*))))).

**Figure 1.**
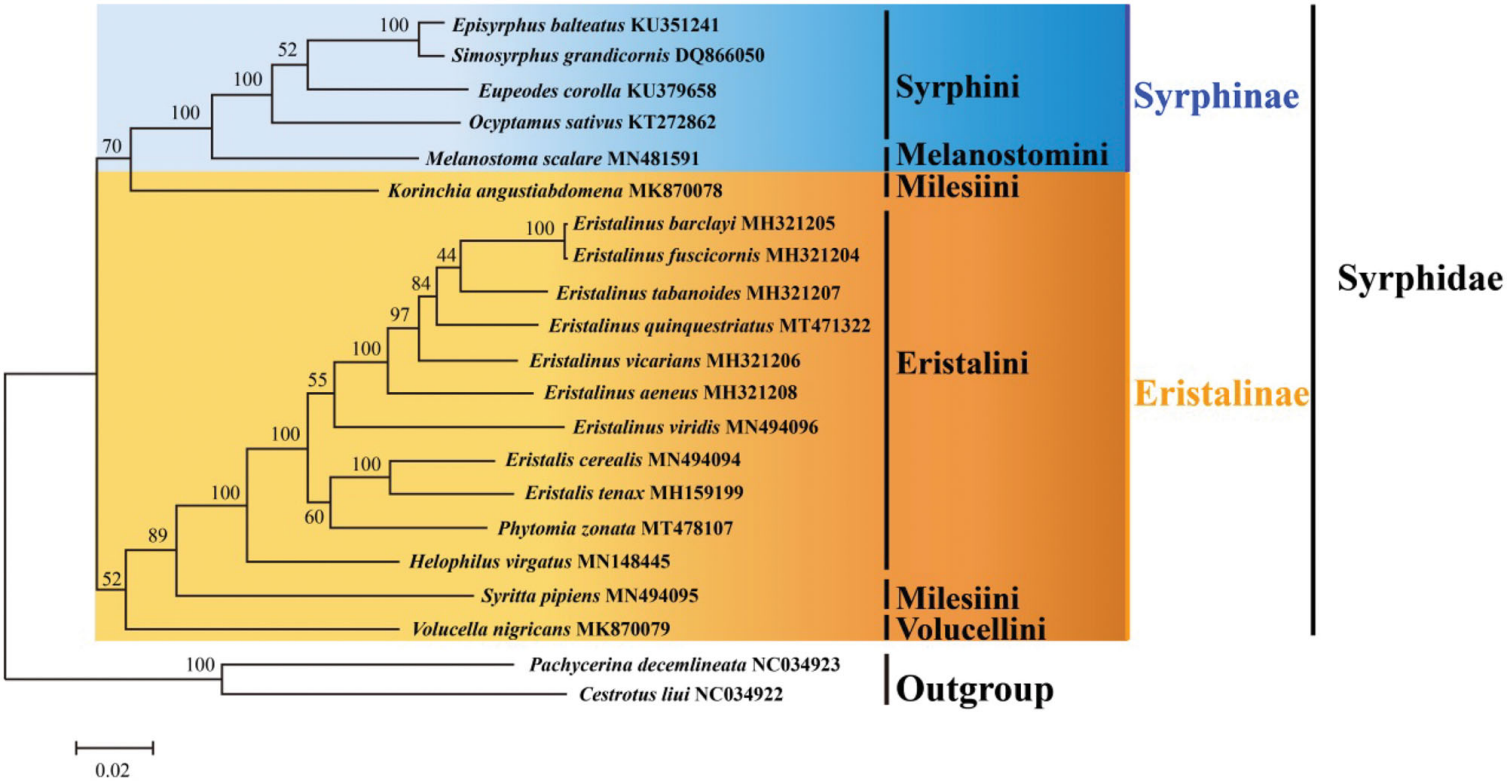
Maximum Likelihood phylogenetic tree of Syrphidae species. The numbers of branches indicate Bootstrap value.

## Data Availability

The data that support the findings of this study are openly available in GenBank at https://www.ncbi.nlm.nih.gov/genbank/, reference number MT478107.
